# Co-existence of ANCA–associated vasculitides with immune-mediated diseases: a single-center observational study

**DOI:** 10.1007/s00296-024-05631-3

**Published:** 2024-06-25

**Authors:** Anna Masiak, Ewa Jassem, Alicja Dębska-Ślizień, Barbara Bułło-Piontecka, Bożena Kowalska, Michał Chmielewski

**Affiliations:** 1 Department of Rheumatology, Clinical Immunology, Geriatrics and Internal Medicine, Medical University of Gdansk, Gdańsk, Poland; 2 Department of Pulmonology and Allergology, Medical University of Gdansk, Gdańsk, Poland; 3 Department of Nephrology, Transplantology and Internal Diseases, Medical University of Gdansk, Gdańsk, Poland; 4 Department of Otolaryngology, Medical University of Gdansk, Gdańsk, Poland

**Keywords:** ANCA-associated vasculitis, Polyautoimmunity

## Abstract

**Background:**

Antineutrophil cytoplasmic antibody-associated vasculitides (AAV) is a group of systemic necrotizing small vessel autoimmune diseases, with microscopic polyangiitis (MPA) and granulomatosis with polyangiitis (GPA) being the two most common. The co-existence of AAV with different immune-mediated diseases (autoimmune disesases - AID) might affect the clinical presentation of the primary disease. The purpose of the study was to assess the co-existence of AAV with AID and to investigate whether it affects the characteristics and the course of AAV.

**Methods:**

A retrospective single-center study was performed to identify patients with a diagnosis of MPA or GPA and concomitant AID, and to investigate their clinical features and characteristics. The group consisted of consecutive unselected AAV patients treated at a large university-based hospital, since 1988 with follow-up until 2022.

**Results:**

Among 284 patients diagnosed either with GPA (232) or MPA (52), 40 (14,1%) had co-existing AIDs. The most frequent were: Hashimoto thyroiditis (16 cases), rheumatoid arthritis (8 cases), followed by psoriasis (6 cases), pernicious anemia (3 cases), and alopecia (3 cases). Patients with autoimmune comorbidities had a significantly longer time between the onset of symptoms and the diagnosis (26 vs. 11 months, *p* < 0.001). Laryngeal involvement (20.0% vs. 9.0%, *p* = 0,05), peripheral nervous system disorders (35.0% vs. 13.9%, *p* < 0.001), and neoplasms (20.0% vs. 8.6%, *p* = 0,044) were more common in patients with AID comorbidities, compared to subjects without AID. In contrast, renal involvement (45.0% vs. 70.9%, *p* = 0.001) and nodular lung lesions (27.5% vs. 47.5%, *p* = 0.044) were significantly less frequent in patients with co-morbidities. Following EUVAS criteria, patients with autoimmune co-morbidities had a generalized form of the disease without organ involvement (52.5% vs. 27.2%, *p* = 0.007), while the others had a higher percentage of generalized form with organ involvement (38.3% vs. 20.0%, *p* = 0.007).

**Conclusions:**

The coexistence of AAV with different autoimmune diseases is not common, but it might affect the clinical course of the disease. Polyautoimmunity prolonged the time to diagnosis, but the AAV course seemed to be milder. Particular attention should be paid to the increased risk of cancer in these patients. It also seems reasonable that AAV patients should receive a serological screening to exclude the development of overlapping diseases.

## Introduction

Antineutrophil cytoplasmic antibody-associated vasculitides (AAV) are a group of systemic necrotizing small vessel diseases comprising microscopic polyangiitis (MPA), granulomatosis with polyangiitis (GPA) and eosinophilic granulomatosis with polyangiitis (EGPA). Those are rare diseases with an estimated prevalence of 42.1/100,000 [1–3]. GPA and MPA are more frequent than EGPA. In a study by Berti et al. the annual incidence rates per 100 00 population of GPA, MPA, and EGPA y were 1.3 (95% CI 0.8–1.8), 1.6 (95% CI 1.0–2.2), and 0.4 (95% CI 0.1–0.6), respectively [1]. AAV are characterized by a chronic course with a broad spectrum of symptoms. The respiratory tract and kidneys are the most common localization of lesions, although vasculitis can affect almost any organ and cause life-threatening organ damage. The pathogenesis of AAV is complex and not fully specified. It is believed that both genetic, epigenetic, and environmental factors contribute to the etiology and pathogenesis of AAV [4]. It was demonstrated that about 20% of AAV risk is due to genetic factors [5].

Autoimmune diseases (AID) are a group of heterogeneous diseases, but they share similar clinical manifestations, that are often difficult to differentiate [6]. The link between various AID, both organ-specific and systemic, is well known [7]. The overlapping syndromes of some AID are quite common and well described, including the co-occurrence of systemic lupus erythematosus (SLE) with rheumatoid arthritis (RA) or Sjögren’s syndrome, or coexistence of SLE, thyroid disease, Sjögren’s syndrome and antiphospholipid syndrome [8, 9]. The coincidence of more than one AID in a single patient is referred to as ‘polyautoimmunity’ [8]. When three or more autoimmune diseases coexist, this condition is called multiple autoimmune syndrome (MAS) [10]. Although the exact pathogenic mechanism of the coexistence of AID has not been clearly defined, genetic and environmental factors, immune defects or hormonal changes, may play a key role in polyautoimmunity [8, 11]. Polyautoimmunity in AAV patients’ is not fully elucidated [12]. There are case reports of patients with AAV who have been diagnosed with other than AAV AID including RA [13], SLE [14], Sjögren’s syndrome [15] and scleroderma [16]. Martín-Nares in his publication [12] described the link between AAV and other immune-mediated conditions.

It is still unclear whether the coexistence of AAV with other autoimmune diseases has any impact on their clinical presentation, course, response to treatment or complications.

The purpose of the study was to assess the prevalence of the co-existence of AID with the two most common antineutrophil cytoplasmic AAV- GPA and MPA, and to establish the clinical features and characteristics of AAV patients affected by polyautoimmunity.

## Materials and methods

A retrospective single-center study was performed to identify patients with a diagnosis of MPA or GPA, and concomitant autoimmune diseases. The group consisted of consecutive unselected patients treated at a large university-based hospital between 1988 and 2022. Diagnosis of GPA (previously Wegener’s granulomatosis) [17] was based on the ACR classification criteria established in 1990 [18] and definition of the disease established during the Chapel Hill Consensus Conference in 1994 [19]. Diagnosis of MPA was based on the Chapel Hill definition [19]. Two patients diagnosed in 1988, one - in 1990 and three - in 1991 had typical histopathological changes and assessed afterwards met the above mentioned criteria.

Patients were followed up from the time of diagnosis to their death or most recent hospital or outpatient’s assessment. Sociodemographic characteristics, symptoms, follow-up time, patients outcome, type of AAV, disease relapses, as well as clinical and serological characteristics of the patients were assessed. The forms of the disease were determined according to the EUVAS criteria as limited (lesions in upper and/or lower respiratory tract, without other organ lesions, and without general symptoms), early systemic (other systemic lesions are present compared to a limited form, but without organ-threatening symptoms or life-threatening symptoms), generalized with organ involvement (symptoms of organ-threatening failure, creatinine level < 5.6 mg/dl) and severe (symptoms of renal failure - creatinine levels > 5.6 mg/dl or other organ failure) [20].

Patient records were analysed for diagnoses of immune-mediated disease other than AAV. Organ-specific as well as systemic diseases were considered. Its presence was confirmed if this diagnosis was included in the list of diagnoses of individual patients performed by attending doctor.

As the study was retrospective in nature, bioethics committee approval was not required.

### Statistical analysis

Data analysis was carried out using IBM SPSS Statistics 28.0. Results are reported as statistically significant when *p* < 0.05. In order to compare the two groups in terms of nominal variables, analysis was performed using either the χ2 test of independence or Fisher’s exact test (if the expected number was less than 5). As a post hoc analysis to determine differences, the Z column proportion test with Bonferroni significance level correction was used. Mann-Whitney U test was used for continuous variables.

## Results

Among 284 patients, 40 (14,1%) had other than AAV autoimmune disease (31/232 with GPA and 9/52 with MPA). The most frequent overlapping AID were Hashimoto thyroiditis (*n* = 16) and rheumatoid arthritis (RA) (*n* = 8), followed by psoriasis (*n* = 6), pernicious anemia (*n* = 3), alopecia areata (*n* = 3) and Sjögren’s syndrome (*n* = 2). Single patients were also diagnosed with primary biliary cirrhosis, polychondritis, autoimmune hepatitis, pyoderma gangrenosum, inflammatory bowel disease, IgA glomerulonephritis, IgG4-related retroperitoneal fibrosis, celiac disease, Graves -Basedow thyroiditis and Takayasu arteritis.

Patients with autoimmune co-morbidities had a significantly longer time from the onset of symptoms to the diagnosis of vasculitis than those without comorbidities (26 vs. 11 months, *p* < 0.001). However, the two groups of patients did not differ in terms of the age at vasculitis diagnosis, the number of relapses or the time until the first relapse. Laryngeal involvement (20.0% vs. 9.0%, *p* = 0,05) and peripheral nervous system disorders (35.0% vs. 13.9%, *p* < 0.001), were more common in patients with AID comorbidities, compared to subjects without AID. In contrast, renal involvement and renal failure (45.0% vs. 70.9%, *p* = 0.001) as well as nodular lung lesions (27.5% vs. 47.5%, *p* = 0.044) were significantly less frequent in patients with co-morbidities. The AID group had a higher incidence of neoplasms (20.0% vs. 8.6%, *p* = 0,044). Among cancers, 2 lymphomas were diagnosed, as well as lung cancer, clear cell renal cell carcinoma, breast cancer, testicular cancer, bladder cancer and skin cancer in single cases. Following EUVAS criteria, patients with autoimmune co-morbidities had a generalized form of the disease without organ involvement (early systemic) (52.5% vs. 27.2%, *p* = 0.007), while the others had a higher percentage of generalized form with organ involvement (38.3% vs. 20.0%, *p* = 0.007). However, the percentage of patients with limited and severe forms of the disease was similar in both groups. Most of the patients without autoimmune co-morbidities were cANCA positive (67.1 vs. 47.5%, *p* = 0.017). No other differences were noted between the groups including basic laboratory results. Results are summarized in Table 1. In terms of treatment, significant differences between the two groups were found for remission during maintenance therapy, whereas methotrexate was used significantly more often in the group of patients with co-morbidities (Table 2).

**Table 1:** Comparative analysis of patients with and without autoimmune co-morbidities in terms of analyzed parameters

Category	Total	Co-existence of autoimmune diseases		p
		No	Yes	
**Disease**, *n (%)*				
GPA	232 (81.7)	201 (82.4)	31 (77.5)	0.460
MPA	52 (18.3)	43 (17.6)	9 (22.5)	
**Sex**, *n (%)*				
Females	148 (52.1)	131 (53.7)	17 (42.5)	0.189
Males	136 (47.9)	113 (46.3)	23 (57.5)	
Age at diagnosis, *n (%)*	284	52.5 (16.44)	50.5 (14.56)	0.351
Age at diagnosis > 60 years, *n (%)*	95 (33.5)	85 (34.8)	10 (25.0)	0.222
Time from the onset of symptoms to the diagnosis (months)	13.71 (30.6)	11.68 (25.0)	26.13 (52.0)	**< 0.001**
Kidney involvement, *n (%)*	191 (67.3)	173 (70.9)	18 (45.0)	**0.001**
Kidney failure, *n (%)*	151 (53.2)	136 (55.7)	15 (37.5)	**0.032**
Need for hemodialysis at diagnosis, *n (%)*	84 (29.6)	77 (31.6)	7 (17.5)	0.071
Kidney transplantation, *n (%)*	17 (6.0)	16 (6.6)	1 (2.5)	0. 316
Upper respiratory tract involvement, *n (%)*	193 (68.0)	168 (68.9)	25 (62.5)	0.425
Nose, *n (%)*	139 (48.9)	121 (49.6)	18 (45.0)	0.590
Throat, *n (%)*	24 (8.5)	18 (7.4)	6 (15.0)	0.124
Larynx, *n (%)*	30 (10.6)	22 (9.0)	8 (20.0)	**0.050**
Sinuses, *n (%)*	118 (41.5)	101 (41.4)	17 (42.5)	0.895
Ear, *n (%)*	85 (29.9)	77 (31.6)	8 (20.0)	0.139
Lungs involvement, *n (%)*	185 (65.1)	164 (67.2)	21 (52.5)	0.070
Nodular/infiltrative lesions in lungs, *n (%)*	127 (44.7)	116 (47.5)	11 (27.5)	**0.018**
Interstitial lung changes, *n (%)*	105 (37.0)	95 (38.9)	10 (25.0)	0.091
Bronchial stenosis, *n (%)*	26 (9.2)	24 (9.8)	2 (5.0)	0.552
Skin involvement, *n (%)*	109 (38.4)	92 (37.7)	17 (42.5)	0.563
Peripheral nervous system involvement, *n (%)*	48 (16.9)	34 (13.9)	14 (35.0)	**< 0.001**
Central nervous system involvement, *n (%)*	43 (15.1)	38 (15.6)	5 (12.5)	0.615
Eye involvement, *n (%)*	83 (29.2)	70 (28.7)	13 (32.5)	0.623
Pseudotumor orbitae, *n (%)*	19 (6.7)	14 (5.7)	5 (12.5)	0.161
Smoking, *n (%)*				
Currently	42 (14.8)	35 (14.3)	7 (17.5)	**0.010**
Past	80 (8.5)	74 (30.3)	6 (15.0)	
Never	138 (48.6)	111 (45.5)	27 (67.5)	
**Disease form**, *n (%)*				
Limited	29 (10.2)	24 (9.9)	5 (12.5)	**0.007**
Early systemic	87 (30.7)	66 (27.2)	21 (52.5)	
Generalized with organ involvement	101 (35.7)	93 (38.3)	8 (20.0)	
Severe	66 (23.3)	60 (24.7)	6 (15.0)	
ANCA negative, *n (%)*	29 (10.2)	22 (9.0)	7 (17.5)	0.152
ANCA positive, *n (%)*	254 (89.4)	221 (90.6)	33 (82.5)	0.160
cANCA, *n (%)*	182 (64.3)	163 (67.1)	19 (47.5)	**0.017**
pANCA, *n (%)*	79 (27.9)	63 (25.9)	16 (40.0)	0.066
Atypical ANCA, *n (%)*	9 (3.2)	6 (2.5)	3 (7.5)	0.119
Number of relapses	1.02 (1.27)	0.99 (1.25)	1.20 (1.44)	0.327
Time to first relaps (months)	29.25 (29.2)	29.54 (30.5)	27.80 (22.0)	0.809
Incidence of thrombotic complications, *n (%)*	43 (15.1)	37 (15.2)	6 (15.0)	0.979
Cancer occurrence, *n (%)*	29 (10.2)	21 (8.6)	8 (20.0)	**0.044**

GPA – granulomatosis with polyangiitis; MPA – microscopic polyangiitis; ANCA - Antineutrophil cytoplasmic antibody

**Table 2:** Comparative analysis of patients with and without autoimmune co-morbidities in terms of the parameters analyzed

Category	Total	Co-existence of autoimmune diseases		*p*
		No	Yes	
Anaemia, *n (%)*	206 (75.2)	179 (76.2)	27 (69.2)	0.353
Thrombocytosis (> 400G/l), *n (%)*	92 (35.1)	80 (35.7)	12 (31.6)	0.621
CRP, *M(SD)*	89.96 (87.49)	93.27 (89.68)	71.65 (72.48)	0.226
**Treatment of remission induction**				
Cyclophosphamide, *n (%)*	206 (72.5)	178 (73.0)	28 (70.0)	0.698
Methotrexate, *n (%)*	29 (10.2)	23 (9.4)	6 (15.0)	0.267
Cyclosporine, *n (%)*	1 (0.4)	1 (0.4)	0 (0)	1.000
Azathioprine, *n (%)*	3 (1.1)	2 (0.8)	1 (2.5)	0.367
Mycophenolate mophetil, *n (%)*	1 (0.4)	1 (0.4)	0 (0)	1.000
Rituximab, *n (%)*	2 (0.8)	0 (0)	2 (0.7)	1.000
**Treatment of remission maintenence**				
Cyclophosphamide, *n (%)*	48 (18.2)	44 (19.6)	4 (10.3)	0.165
Azathioprine, *n (%)*	112 (42.6)	96 (42.9)	16 (41.0)	0.831
Cyclosporine, *n (%)*	6 (2.3)	3 (1.3)	3 (7.7)	0.044
Methotrexate, *n (%)*	56 (21.3)	41 (18.3)	15 (38.5)	**0.005**
Mycophenolate mophetil, *n (%)*	25 (9.5)	22 (9.8)	3 (7.7)	1.000
Rituximab, *n (%)*	56 (19.7)	48 (19.7)	8 (20.0)	0.961

CRP – C-reactive protein

## Discussion

The complex etiopathogenesis of autoimmune diseases involving, inter alia, genetic predisposition and environmental factors, makes polyautoimmunity relatively common. A British population-based study established the incidence and prevalence of 19 autoimmune disorders in a population of more than 22 million individuals [21]. The authors concluded, that autoimmune diseases affect approximately one in ten individuals. They also stated, that their burden continues to increase over time especially for Sjogren’s syndrome and Graves’s disease. The authors also confirmed that autoimmune diseases are commonly associated with each other, in particular Sjögren’s syndrome, systemic lupus erythematosus, and systemic sclerosis [21].

Apart from descriptions of individual cases and case series, there are only single analyses of groups of patients with AAV in the context of polyautoimmunization [12, 22]. Thus, there are insufficient data to assess conclusively whether the phenomenon affects the clinical presentation, course of the disease and prognosis in patients with AAV. Martin-Nares et al. reported that, in 28 (11.3%) of 247 patients, AAV was associated with other AID [12]. Guibert et al. showed, on the basis of the Maine-Anjou AAV Registry, that 15% of 106 patients had a diagnosis of other connective tissue disease [22]. In the presented group of patients with either GPA or MPA, the co-existence of other autoimmune disorders was found in 14% of patients. A similar result compared to the aforementioned patient groups, showing that the overlap syndrome is, in fact, not that uncommon in AAV patients. In the present study, we have confirmed that observation for the Polish population, and demonstrated that co-existing autoimmune diseases might affect the clinical course of AAV. Among our patients, polyautoimmunity was associated with a prolonged time to AAV diagnosis, but the AAV course seemed to be milder with less common renal involvement.

The most prevalent co-morbidity in our group was autoimmune thyroid disease – 16 cases with Hashimoto and 1 with Graves-Basedow thyroiditis. The association of AAV with thyroid disease was also pointed out by other authors. Prendecki et al., in their group of 279 patients with AAV, who found that 60 (21.5%) patients had some forms of thyroid disease [23]. A similar proportion of 20% of thyroid disease among AAV patients with kidney involvement was reported in an American case-control series [24]. A Swedish study reporting co-morbidities in patients with AAV found a slightly lower prevalence of thyroid disease equaling 14.5% [25]. In their study, Pearce et al. showed that, similarly to having rheumatoid arthritis or inflammatory bowel disease, patients with thyroid diseases were almost twice as likely to develop AAV, compared to controls [26]. It was also reported that a history of thyroid disease among patients with GPA was associated with more severe vasculitic symptoms [27]. No such relationship was found in our group of patients. The co-existence of AAV with immune-mediated thyroid disease is a typical example of a genetic predisposition to AID. It has been demonstrated that a polymorphism of the cytotoxic T lymphocyte antigen-4 (CTLA-4) gene is linked to autoimmune thyroiditis [26, 27] as well as to granulomatosis with polyangiitis [28]. Similarly, a functional polymorphism in the protein tyrosine phosphatase gene, the PTPN22 620 W allele, has been recognized as a predisposing factor for several autoimmune diseases including Graves and Hashimoto thyroid diseases [29] the same as for GPA and ANCA positivity [30]. Human thyroid peroxidase (TPO) antibody and MPO have a 44% sequence homology, raising the possibility that cross-reactivity between TPO and MPO is responsible for the increased thyroid disease in patients with AAV [29].

Other autoimmune diseases have also been found in patients with AAV. Among systemic connective tissue disorders, RA followed by Sjögren’s syndrome and scleroderma, mixed connective tissue disease, and SLE, were found to be present [12], while in another evaluation it was RA, followed by systemic sclerosis, Sjögren’s syndrome and polymyalgia rheumatica [22]. In our patients, the most common overlapping rheumatic disease was also RA, followed by Sjögren’s syndrome. The RA and AAV overlap syndromes were described by Draibe and Salama, who presented six patients with rheumatoid arthritis who subsequently developed AAV (mainly MPA), and found in literature another 29 RA and AAV overlap cases [13]. Of these cases, 13 patients had an overlap between RA and GPA, 15 RA and MPA and only 1 with RA and EGPA [13]. Wu et al. described 47 patients (15 from their hospital and 32 from the literature). In most of them, RA preceded the diagnosis of AAV and then manifested itself as microscopic polyangiitis (MPA) with renal involvement [31]. Co-existing RA and AAV requires special attention because disease modifying antirheumatic drugs used for RA may change the typical manifestations of AAV and delay the AAV recognition or even result in poor outcome. Wu et al. [31] and Kurita et al. [32] demonstrated that the co-existence of RA and AAV (mainly MPA) was associated with a lower frequency of rapidly progressive glomerulonephritis, and more chronicity in renal pathology. Finally, as with the association between AAV and thyroid disease, the association of AAV with RA also has a genetic basis. The predispositions to autoimmunity which involve the HLA region or genes such as PTPN22 [33], but also polymorphisms in uteroglobin (a multifunctional protein with anti-inflammatory properties) and NF-κB2 (a transcription factor that regulates the expression of a wide range of immune response genes) were associated with a genetic predisposition towards developing both vasculitis and RA [34].

We found no co-existence of lupus or scleroderma among our AAV patients. While ANCA detection is quite frequent in SLE patients, the association between SLE and AAV seems to be rare. In support, in a nationwide French study, only eight patients with both diseases were identified [35] and only one such patient in another study, by Guibert at al [22]. .

Autoimmune diseases are more common in women [36]. Interestingly, we found no such prevalence pattern in our group, although others described that 75% of AAV patients with AID co-existence were female [22].

An interesting aspect is whether the co-existence of AID with AAV has any impact on the clinical picture and prognosis of patients. The above mentioned study by Guibert et al. [22] found no differences with respect to AAV presentation, including kidney function and histological involvement, but also the age at diagnosis, AAV phenotype, ANCA subtype distribution, organ involvement, or disease activity between AID patients and control patients. Their main finding was that, despite a higher rate of non-renal AAV relapses and venous thrombosis, AID patients seemed to have a comparable prognosis according to a composite criterion, including major events (deaths, severe infections, cancer, cardiovascular events and thrombotic events).

In our group of patients, those with co-existing AID had a longer time to diagnosis, but milder forms of AAV and were less likely to have cANCA positivity. They also had less frequent renal involvement and more frequent upper respiratory tract involvement. Interestingly, the opposite was observed in the group of Prendecki et al. [23]. This may be partly explained by the higher proportion of patients with MPO-ANCA in comparison to our group with a predominance of patients with GPA. Similarly, Martin-Nares also found more patients with concomitant autoimmune systemic diseases among renal-limited vasculitis cases [12].

We did not find a higher incidence of thrombotic complications among our patients. Such a relationship was demonstrated by Guibert at al [22]. in their study.

A very significant finding in our analysis is the higher proportion of neoplasms among patients with co-existing AID. We found no prevalence of any particular cancer, but no such relationship was found in other studies [22]. The association between cancer and autoimmune diseases has been the subject of interest for many years. Patients diagnosed with rheumatoid arthritis [37], SLE [38], systemic sclerosis [39] or primary Sjögren’s syndrome [40] have a considerably increased overall cancer risk with reported standardized incidence ratios (SIR. i.e. a ratio indicating the risk of patients compared with that of the sex-matched, age-matched, and calendar year matched general population) of 1.1–1.5 [41]. On the other hand, some malignancies may present with clinical features resembling an autoimmune disorder and may also cause autoimmunity through various mechanisms [40]. Several studies have consistently demonstrated an increased risk of cancer in GPA with standardized incidence ratios from 1.6 to 3.8 [41–44]; compared to the general population [45, 46]. Meta-analysis performed by Shang et al. indicated that AAV patients treated with cyclophosphamide are at increased risk of late-occurring malignancies, in particular non-melanoma skin cancer, leukemia and bladder cancer [43].

Our study has limitations. This was a retrospective single-center analysis and, as such, precludes conclusions on the causal character of the observed associations. The diagnosis of AID was established by the treating physician and was not further verified by the authors. The strength of the study is its long follow-up, with a median time of 93 months for GPA and 62 months for MPA patients.

## Conclusion

The coexistence of AAV with other autoimmune diseases is not common, but it might have an impact on the clinical course. Polyautoimmunity prolonged the time to diagnosis, but the AAV course seemed to be milder. The observations demonstrated in our group ought to be confirmed in larger groups of patients. Particular attention should be paid to the increased risk of cancer in patients with co-existing autoimmune disorders. It also seems reasonable that AAV patients should be subject to periodic evaluation for new manifestations and should receive a serological screening to exclude the development of overlap syndromes.

## Data Availability

All data generated or analyzed during this study are included in this published article.
